# Effects of Multidisciplinary Rehabilitation Program in Patients with Long COVID-19: Post-COVID-19 Rehabilitation (PCR SIRIO 8) Study

**DOI:** 10.3390/jcm12020420

**Published:** 2023-01-04

**Authors:** Małgorzata Ostrowska, Alicja Rzepka-Cholasińska, Łukasz Pietrzykowski, Piotr Michalski, Agata Kosobucka-Ozdoba, Małgorzata Jasiewicz, Michał Kasprzak, Jacek Kryś, Aldona Kubica

**Affiliations:** Collegium Medicum, Nicolaus Copernicus University, 85-094 Bydgoszcz, Poland

**Keywords:** long COVID-19, post-COVID-19 syndrome, multidisciplinary rehabilitation, physical training, cardioplumonary exercise testing

## Abstract

Up to 80% of COVID-19 survivors experience prolonged symptoms known as long COVID-19. The aim of this study was to evaluate the effects of a multidisciplinary rehabilitation program in patients with long COVID-19. The rehabilitation program was composed of physical training (aerobic, resistance, and breathing exercises), education, and group psychotherapy. After 6 weeks of rehabilitation in 97 patients with long COVID-19, body composition analysis revealed a significant decrease of abdominal fatty tissue (from 2.75 kg to 2.5 kg; *p* = 0.0086) with concomitant increase in skeletal muscle mass (from 23.2 kg to 24.2 kg; *p* = 0.0104). Almost 80% of participants reported dyspnea improvement assessed with the modified Medical Research Council scale. Patients’ physical capacity assessed with the 6 Minute Walking Test increased from 320 to 382.5 m (*p* < 0.0001), the number of repetitions in the 30 s Chair Stand Test improved from 13 to 16 (*p* < 0.0001), as well as physical fitness in the Short Physical Performance Battery Test from 14 to 16 (*p* < 0.0001). The impact of fatigue on everyday functioning was reduced in the Modified Fatigue Impact Scale from 37 to 27 (*p* < 0.0001). Cardiopulmonary exercise test did not show any change. The multidisciplinary rehabilitation program has improved body composition, dyspnea, fatigue and physical capacity in long COVID-19 patients.

## 1. Introduction

Due to the widespread of severe acute respiratory syndrome coronavirus 2 (SARS-CoV-2) and the unpredictable course of the coronavirus disease-2019 (COVID-19), it has become one of the greatest medical challenges of our times [1,2]. Some people infected with the SARS-CoV-2 will remain asymptomatic, some will experience mild to moderate respiratory tract illness, some will become seriously ill, and, finally, some of them will die [3]. Since the beginning of the COVID-19 pandemic, the World Health Organization (WHO) has reported more than 636 million of COVID-19 cases and 6,6 million deaths [4]. However, surviving COVID-19 is only the top of an iceberg. In a substantial proportion of COVID-19 survivors, symptoms endure for months, or new symptoms develop after the acute phase of the disease [5,6,7]. According to the WHO, post-COVID-19 syndrome, also known as long COVID-19, is defined as a constellation of long-term symptoms (lasting for at least 2 months) occurring within 3 months from the onset of COVID-19 [8]. Most commonly reported symptoms of the post-COVID-19 condition include: chest pain, fatigue, dyspnea, cough, and sputum production [9]. However, long COVID-19 is not limited to symptoms from the respiratory tract; it can involve multiple organs and affect many systems [10]. Thus, other symptoms described in the literature encompass: joint pain, myalgia, headache, palpitations, anosmia, dysgeusia, hair loss, and cognitive symptoms such as memory or concentration deficits [6,11,12,13]. Moreover, many psychological problems, including anxiety, depression, and sleep disturbances were reported in post COVID-19 patients [14,15,16]. Factors potentially associated with development of the post-COVID-19 condition include: old age, female sex, severe clinical status, multiple comorbidities, hospital admission, and oxygen supplementation [9]. Nonetheless, long COVID-19 can affect patients independently of the severity of the acute phase of COVID-19, including younger adults and children [11].

The role of physical activity and therapeutic education has been well established in prevention and treatment of many chronic diseases [17,18,19,20,21]. Prolonging hospitalization or self-isolation at home in COVID-19 patients can lead to muscle deconditioning, which may be worsened by a sedentary lifestyle in patients with long COVID-19 who experience persisting symptoms [22,23].

We hypothesized that a multidisciplinary rehabilitation program composed of physical activity, therapeutic education, and psychotherapy sessions can improve symptoms in patients with long COVID-19. The aim of this study was to objectively evaluate the effects of the multidisciplinary rehabilitation program implemented in patients with post-COVID-19 syndrome.

## 2. Materials and Methods

### 2.1. Study Design

The study was designed as a prospective, observational, single-center study involving patients suffering from post-COVID-19 syndrome. Patients experiencing prolonged symptoms lasting for at least 2 months, occurring within 3 months from the onset of COVID-19, admitted to the post-COVID-19 rehabilitation outpatient clinic were recruited in the study. During the initial medical and physiotherapeutic visit, patients were screened for eligibility to participate in our multidisciplinary rehabilitation program as described in our previous publication [24]. Each patient provided written informed consent to participate in the study. The study was conducted in accordance with the Declaration of Helsinki and was approved by the Local Ethics Committee (study approval reference number KB 414/2021). The inclusion criteria: patients experiencing symptoms lasting for at least 2 months, occurring within 3 months from the onset of COVID-19 and up to 12-months post COVID-19 diagnosis, with any of the following were included in the study: (i) at least slight functional limitations diagnosed with the use of the Post-COVID-19 Functional Status (PCFS) scale (score > 1); (ii) decrease of muscle strength according to the Medical Research Council (MRC) scale for muscle strength (score < 5); (iii) severity of dyspnea according to the modified MRC (mMRC) dyspnea scale (score > 2). The exclusion criteria: patients were not eligible to participate in the study if the severity of symptoms prevented them from functioning independently in the physical and/or mental sphere. Baseline clinical evaluation was composed of: body composition assessment, cardiopulmonary exercise testing (CPET), and basic laboratory tests. The following scales were used in baseline physiotherapeutic assessment: dyspnea assessment (mMRC dyspnea scale, score 0–4; Modified Borg scale, score 1–10), fatigue assessment (Modified Fatigue Impact Scale—MFIS, score 0–84), assessment of exercise tolerance (6 Minute Walking Test—6MWT; 30 s Chair Stand Test—30CST), physical fitness assessment (Short Physical Performance Battery test—SPPB test, score 0–12). The multidisciplinary outpatient rehabilitation program was scheduled for six consecutive weeks. It contained: physical training (aerobic, resistance, and breathing exercises; 90 min, 3 times a week), education (30 min, 3 times a week) and group psychotherapy (30 min, once a week) [24]. Additionally, individual psychotherapy, education, or medical assistance was available throughout the rehabilitation period. Closing evaluation included: body composition assessment, the CPET, and physiotherapeutic assessment according to the abovementioned scales.

### 2.2. End Points

The study endpoints assessed after completion of the 6 week multidisciplinary rehabilitation program included:

- change in body composition;
- change in the CPET results (peak oxygen consumption [VO_2 peak_], predicted VO_2 peak_, VO_2peak_/breathing frequency_peak_, predicted VO_2peak_/breathing frequency_peak_ (%), minute ventilation to carbon dioxide production [VE/VCO_2_], oxygen uptake efficiency slope [OUES], anaerobic threshold [AT]);
- change in perceived fatigue accordingly to the MFIS;
- change in perceived dyspnea accordingly to the mMRC dyspnea scale and the Borg scale;
- change in exercise tolerance accordingly to the 6MWT and the 30 CST;
- change in physical fitness accordingly to the SPPB test.

### 2.3. Statistical Analysis

The statistical analysis was carried out using the Statistica 13.0 package (TIBCO Software Inc, Palo Alto, CA, USA). Categorical variables were expressed as the number and the percentage. The Shapiro–Wilk test demonstrated non-normal distribution of the investigated continuous variables. Therefore, continuous variables were presented as medians with interquartile range and non-parametric tests were used for statistical analysis. Comparisons between continuous parameters pre- and post-rehabilitation were performed with the Wilcoxon signed-rank test. Results were considered significant at ***p*** < 0.05.

## 3. Results

Between 1 June 2021 and 31 July 2022, a total of 97 consecutive patients with post-COVID-19 syndrome were enrolled into the study. The majority of patients suffered from slight functional limitations according to the PCFS scale (PCFS = 2 in 93.8% of patients). Decrease of muscle strength according to the MRC scale (MRC < 5) was recorded in 6.2% of patients. Significant dyspnea was found in 23.7% of study participants (mMRC > 2). Baseline characteristics of the study population are presented in Table 1.

**Figure 1:**
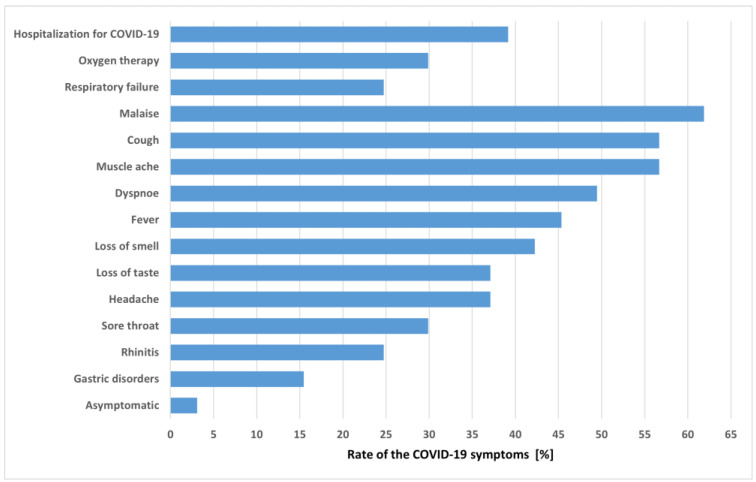
The COVID-19 symptoms.

The majority of patients was previously fully vaccinated (two doses) against COVID-19 (76.3%). The most frequently reported adverse reactions post vaccination were pain in the area of injection (60.8%) and malaise (33.8%). During the acute phase of COVID-19, 39.2% of enrolled patients were hospitalized and 24.7% presented with respiratory failure. Patients most frequently complained of malaise, cough, and muscle ache (Figure 1).

**Figure 2:**
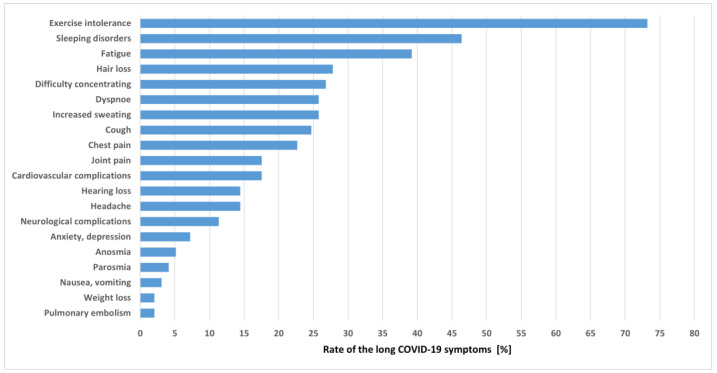
Long COVID-19 symptoms.

After the acute phase of COVID-19, the majority of patients reported exercise intolerance (73.2%), followed by sleep disturbances (46.9%) and fatigue (39.2%). The full list of the recorded long COVID-19 symptoms is shown in Figure 2.

After six weeks of physical training, the body composition analysis revealed a significant decrease of abdominal fatty tissue mass (from 2.8 kg to 2.5 kg; *p* = 0.0086), waist circumference (from 94 cm to 93 cm; *p* = 0.0008), and cell hydration (from 84.2% to 80.3%; *p* = 0.0001), with concomitant increase in skeletal muscle mass (from 23.2 kg to 24.2 kg; *p* = 0.0104) and phase angle (from 4.8 to 5.0; *p* < 0.0001) (Table 2).

Out of 97 study participants, CPET was not performed in 57 patients due to the severity of symptoms prior to rehabilitation. Therefore, CPET was performed only in 40 patients (41.2%) before starting the physical training program and after finishing the program. The median peak oxygen consumption (VO_2peak_) value has not changed, accounting for 18 mL/kg/min (*p* = 0.74); however, a slight increase in the median percentage of predicted peak VO_2_ was noted, accounting for 79.5% before and 81.0% after completing the program (*p* = 0.86; Table 2). The median minute ventilation to carbon dioxide production (VE/VCO_2_ slope) decreased from 29.4 to 29.2 after completing the physical training (*p* = 0.81; Table 2). The median percentage of predicted anaerobic threshold had not changed, accounting for 50% (*p* = 0.99; Table 2).

According to the Modified Borg Scale (1–10), which is a valid and reliable assessment tool for dyspnea, the reported median value changed from 3 (i.e., moderate dyspnea) to 2 (i.e., slight dyspnea) after completing the rehabilitation program (*p* < 0.0001; Table 3). Similarly, significant improvement of the perceived dyspnea was recorded with the use of the mMRC dyspnea scale, with patients claiming to walk slower than other people of same age on the level due to shortness of breath or need to stop for breath when walking at own pace (median mMRC = 2) before entering the study, and feeling breathless when hurrying on the level or walking up a slight hill (median mMRC = 1) after completing the study (*p* < 0.0001; Table 3).

Since fatigue was the third most frequently reported long COVID-19 symptom, the MFIS scale was used to assess its impact on everyday functioning. The median MFIS score reduced from 37 at baseline to 27 after completing the rehabilitation program (Table 3).

Patients’ physical capacity assessed with the 6MWT increased from a median value of 320 m before starting the physical training program to 382.5 m after completing the program (*p* < 0.0001; Table 3). Similarly, in the 30CST, the median value of chair stand repetitions was 13 before entering the rehabilitation program, and increased to 16 after finishing the program (*p* < 0.0001; Table 3). Moreover, the SPPB test assessing gait speed, chair stand, and balance revealed an increase in the median value from 14 to 16 (maximal score) after 6 weeks of physical training (*p* < 0.0001; Table 3).

**Figure 3:**
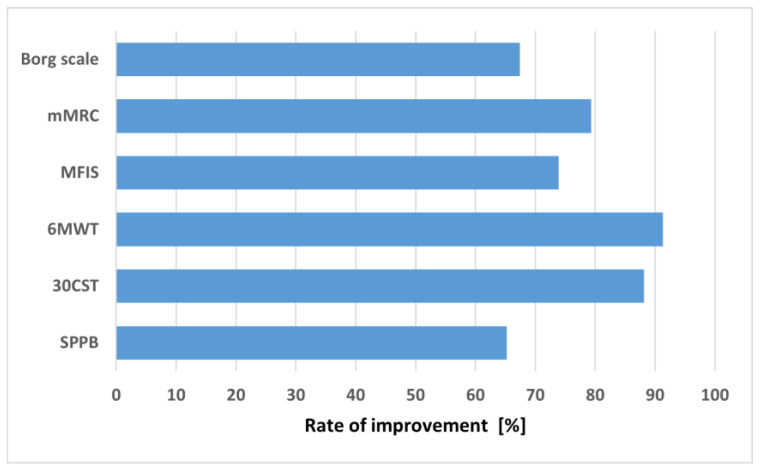
The percentage of patients in whom an improvement was noted after completing the rehabilitation program. Abbreviations: 30CST, 30 s Chair Stand Test; MFIS, Modified Fatigue Impact Scale; mMRC, modified Medical Research Council; 6MWT, 6 Minute Walking Test; SPPB, Short Physical Performance Battery Test.

According to the scales used, a significant improvement of dyspnea, fatigue, and physical capacity was noted in vast majority of study participants (Figure 3).

## 4. Discussion

Up to 80% of 636 million patients with confirmed infection with SARS-CoV-2 virus may experience prolonged symptoms known as long COVID-19 [8,9]. We performed this study to objectively evaluate the outcomes of the multidisciplinary rehabilitation program implemented in patients with post-COVID-19 syndrome. We found that a multidisciplinary rehabilitation program composed of physical activity, therapeutic education, and psychotherapy sessions can improve: (1) body composition; (2) dyspnea; (3) fatigue; and (4) physical capacity in majority of patients with long COVID-19.

Long COVID-19 affects millions of people worldwide, decreasing their quality of life and having impact on whole societies [8,9]. There is no specific pharmaceutical treatment designated to relieve the long COVID-19 symptoms [11].

In a cross-sectional survey conducted in Israel, Kuodi et al. postulated that vaccination with at least two doses of COVID-19 vaccine reduces the risk of developing any of the long COVID-19 symptoms [25]. Similar findings of decreased risk of long COVID-19 in double vaccinated individuals were reported in a prospective, community-based, nested, case control study from the UK including 1,240,009 smartphone app users, of whom 6030 (0.5%) tested positive for SARS-CoV2 after their first vaccination, and 2370 (0.2%) after their second vaccination [26]. In our study 76.3% of participants had received two doses of vaccination against COVID-19.

In March 2022, in Great Britain, the National Institute for Health and Care Excellence, in collaboration with the Scottish Intercollegiate Guidelines Network and the Royal College of General Practitioners, developed the COVID-19 rapid guideline: managing the long-term effects of COVID-19 [27]. The panel encouraged individualized, multidisciplinary rehabilitation in patients with long COVID-19, covering physical, psychological, and psychiatric support in order to restore functioning, health, and wellbeing. The Standford Hall consensus statement for post-COVID-19 rehabilitation underlined the importance of a patient-centered holistic approach, tailored to individual needs, with education playing a key role [28]. The rehabilitation should be aimed at relieving dyspnea, psychological distress, and improving participation in rehabilitation, physical function, and quality of life, with participants being reviewed throughout the process. In our study, a comprehensive medical and physiotherapeutic assessment was performed to individualize the multidisciplinary rehabilitation program, composed of physical training, therapeutic education, and individual psychotherapy.

The first report on the favorable impact of respiratory rehabilitation in elderly patients diagnosed with COVID-19 came from an observational, prospective study by Liu et al. [29]. The study included 72 participants, of whom 36 underwent 6 weeks of respiratory rehabilitation, leaving the rest without any intervention. They found the 6 week respiratory rehabilitation improved respiratory function (measured with plethysmography and diffusing lung capacity for carbon monoxide), distance in 6MWT, quality of life (assessed with SF-36 score), and anxiety (assessed with Self-Rating Anxiety Scale). A retrospective study on early rehabilitation in 28 patients hospitalized for COVID-19 introduced as soon as they were hemodynamically and respiratory stable showed improvement in maximal distance in 6MWT and feeling thermometer after 2–4 weeks of individualized exercise training [30]. We also found the 6 week rehabilitation program to improve maximal distance in 6MWT together with an increased number of repetitions in 30CST and overall fitness in SPPB.

A case report on a Japanese man in his 40s who underwent severe COVID-19 infection demonstrated rehabilitation therapy based on exercise to improve gait, together with trunk and lower limb muscle strength. It increased psoas muscle volume with a concomitant decrease in whole-body extracellular water on a computed tomography scan [31]. We also observed favorable changes in body composition after 6 weeks of physical training including a decrease of abdominal fatty tissue mass, waist circumference, and cell hydration, with concomitant increase in skeletal muscle mass and phase angle. A study by Udina C, et al. assessed the impact of multi-component therapeutic exercise in 33 post-COVID-19 patients [32]. The 30 min 7 days/week therapeutic exercise program composed of resistance, endurance, and balance training improved the functional status of COVID-19 survivors, as measured with the SPPB test and the 6MWT.

The only available report on effects of exercise rehabilitation in patients with long COVID-19 comes from Italy [33]. Barbara et al. included 50 patients with long COVID-19 and reduced exercise capacity, defined as predicted peak oxygen consumption <85% in baseline CPET performed 3 months post COVID-19. All participants underwent a laboratory controlled 8-week aerobic and resistance exercise training program with exercise sessions 3 times a week. The VO_2peak_ increased from 17.8 to 20.5 mL/kg/min (*p* < 0.001), with a concomitant increase of predicted VO_2_ from 66.5% to 80.7% (*p* < 0.001). The mean VE/VCO_2_ slope decreased from 36.9 to 32.7 (*p* = 0.013). The respiratory exchange ratio did not change, accounting for 1.1 (*p* = 0.835). The CPET results were in line with our observations, however, in our study, the differences were statistically insignificant. The effects of the resistance training showed a significant increase in the number of the maximum value of repetitions of push ups, pull downs, leg extensions and flexions, abductions and adductions, leg press, abdomen, and back. We observed an improvement in physical fitness in validated scores such as the 30 s Chair Stand Test and the Short Physical Performance Battery test.

Other trials examining the effects of rehabilitation programs in long COVID-19 patients are currently ongoing [34,35]. Due to the growing number of patients with long COVID-19, there is an urgent need to create safe and effective rehabilitation programs to relieve symptoms and promote early return to work and social roles. An interesting alternative of Virtual Rehabilitation Program for long COVID-19 patients was proposed by Flannery et al. [36]. A 10-week Virtual Rehabilitation Program aimed to provide early education and self-management techniques to relieve the most common long COVID-19 symptoms. The virtual rehabilitation program was highly valued by the attendees, especially the sessions focusing on key symptoms such as breathlessness and fatigue, as well the opportunity to share their stories and experiences. The main barriers to attendance included use of technology, maintaining a work/life balance, and health inequalities.

The main limitation of our study is that less than a half of the included patients had CPET before and after finishing the rehabilitation program. The optimal intensity of the exercise training was individualized accordingly to patients’ physical capacity, which limits generalization of the obtained results. Patients were enrolled to the rehabilitation program at different time points post SARS-CoV-2 infection.

## 5. Conclusions

A multidisciplinary rehabilitation program composed of physical training, education, and psychotherapy in patients with long COVID-19 has improved: (i) body composition—increase in skeletal muscle mass and reduction of fat; (ii) dyspnea—according to the Borg scale and the mMRC dyspnea scale; (iii) fatigue—with a decrease in the MFIS scale; and (iv) physical capacity—by significant increase in walking distance, gait speed, and chair stand as assessed with the 6MWT, the 30CST, and the SPPB.

## Figures and Tables

**Table 1:** Baseline characteristics of study participants.

Variable	Overall Study Population (*n* = 97)
Age [years]	60.0 (50.0–68.0)
Female	53 (54.6)
Body mass index [kg/m^2^]	28.1 (24.4–31.1)
Current smoker	11 (11.3)
Hypertension	45 (46.4)
Hyperlipidemia	24 (27.7)
Coronary artery disease	18 (18.6)
Heart failure	6 (6.2)
COPD	12 (12.4)
LVEF [%]	60.0 (55.0–62.0)
Hemoglobin [g/dL]	13.8 (12.9–14.7)
Glucose [mg/dL]	97.0 (90.0–111.0)
eGFR [mL/min]	90.9 (77.5–103.8)
C-reactive protein [mg/L]	1.8 (0.7–4.0)
NT-pro BNP [ng/L]	105.0 (42.5–240.0)
Vaccination against COVID-19	74 (76.3)

Variables are presented as median (interquartile range) or number (%). COPD, chronic obstructive pulmonary disease; COVID-19, coronavirus disease 2019; eGFR, estimated glomerular filtration rate; LVEF, left ventricular ejection fraction; NT-pro BNP, N-terminal pro-brain natriuretic peptide.

**Table 2:** Effects of multidisciplinary rehabilitation program on body composition and cardiorespiratory fitness.

	Pre	Post	*p*
Body composition			
Weight (kg)	78.3 (67.8–98.9)	79.2 (68.2–92.4)	0.6809
BMI (kg/m^2^)	27.4 (24.5–30.9)	27.6 (24.6–30.7)	0.5114
Waist circumference (cm)	94.0 (88.0–105.0)	93.0 (88.0–107.0)	0.0008
Abdominal fatty tissued mass (kg)	2.8 (1.9–3.9)	2.5 (1.6–3.7)	0.0086
Skeletal muscle mass (kg)	23.2 (18.6–29.3)	24.2 (19.0–30.5)	0.0104
Fat-free body mass (kg)	50.7 (42.0–62.0)	51.7 (42.6–63.3)	0.2428
Fat-free body mass ratio (%)	65.9 (59.4–70.8)	66.3 (57.5–71.6)	0.3916
Body fat mass (kg)	27.5 (19.6–32.9)	27.7 (19.9–33.3)	0.8296
Body fat mass ratio (%)	34.0 (29.2–40.6)	33.7 (28.8–42.5)	0.4471
Body water (kg)	37.5 (31.5–45.6)	38.2 (31.7–46.3)	0.3677
Body water ratio (%)	48.6 (43.1–51.8)	48.8 (43.4–53.0)	0.2394
Extracellular body water (kg)	17.0 (14.8–20.2)	16.9 (14.6–20.4)	0.2228
Extracellular body water ratio (%)	21.7 (20.1–23.1)	21.5 (20.4–23.1)	0.3710
Hydration (%)	84.2 (77.0–90.9)	80.3 (76.4–89.7)	0.0001
Phase angle	4.8 (4.4–5.2)	5.0 (4.6–5.4)	<0.0001
CPET			
VO_2peak_ (mL/kg/min)	18.0 (15.0–18.5)	18.0 (15.5–20.0)	0.7364
Predicted VO_2peak_ (%)	79.5 (70.5–93.0)	81.0 (72.0–92.5)	0.8563
VO_2peak_/breathing frequency_peak_	29.5 (26.0–35.5)	29.5 (25.5–33.5)	0.8563
Predicted VO_2peak_/breathing Frequency_peak_ (%)	100.5 (86.5–124.5)	101.5 (89.5–114)	0.3803
VE/VCO_2_ slope	29.4 (27.6–32.3)	29.2 (24.7–33.5)	0.0806
OUES slope	2.05 (1.4–2.35)	2.1 (1.6–2.5)	0.8512
Predicted AT (%)	50.0 (42.0–59.0)	50 (43.0–60.0)	0.9935

Variables are presented as median (interquartile range). Abbreviations: AT, anaerobic threshold; BMI, body mass index; CPET, cardiopulmonary exercise testing; OUES, oxygen uptake efficiency slope; Pre, before entering the rehabilitation program; Post, after completing the rehabilitation program; VE, minute ventilation; VCO2, carbon dioxide production; VO2peak, peak oxygen consumption. Results were significant at *p* < 0.05.

**Table 3:** Effects of multidisciplinary rehabilitation program assessed with the use of validated scales.

	Pre	Post	*p*
Borg scale	3 (3–5)	2 (1–3)	<0.0001
mMRC	2 (2–2)	1 (0–2)	<0.0001
MFIS	37.0 (26.0–51.0)	27.0 (20.0–36.5)	<0.0001
6MWT	320 (290–380)	382.5 (331.5–435.0)	<0.0001
30CST	13 (10–16)	16 (14–20)	<0.0001
SPPB	14 (13–15)	16 (14–16)	<0.0001

Variables are presented as median (interquartile range). Abbreviations: 30CST, 30 s Chair Stand Test; MFIS, Modified Fatigue Impact Scale; mMRC, modified Medical Research Council; 6MWT, 6 Minute Walking Test; Pre, before entering the rehabilitation program; Post, after completing the rehabilitation program; SPPB, Short Physical Performance Battery Test. Results were significant at *p* < 0.05.

## Data Availability

Not applicable.

## References

[1] Dzieciatkowski T., Szarpak L., Filipiak K.J., Jaguszewski M., Ladny J.R., Smereka J. (2020). COVID-19 challenge for modern medicine. Cardiol. J..

[2] Gajda R. (2022). The fear of COVID—A factor affecting the functioning of emergency medical service. Med. Res. J..

[3] Kowalik M.M., Trzonkowski P., Łasińska-Kowara M., Mital A., Smiatacz T., Jaguszewski M. (2020). COVID-19—Toward a comprehensive understanding of the disease. Cardiol. J..

[4] Coronavirus Disease 2019 (COVID-19): Global Situation, Number of Confirmed Cases. https://covid19.who.int/.

[5] Carfì A., Bernabei R., Landi F. (2020). Gemelli Against COVID-19 Post-Acute Care Study Group. Persistent Symptoms in Patients After Acute COVID-19. JAMA.

[6] Sykes D.L., Holdsworth L., Jawad N., Gunasekera P., Morice A.H., Crooks M.G. (2021). Post-COVID-19 Symptom Burden: What is Long-COVID and How Should We Manage It?. Lung.

[7] Mandal S., Barnett J., Brill S.E., Brown J.S., Denneny E.K., Hare S.S., Heightman M., Hillman T.E., Jacob J., Jarvis H.C. (2021). ARC Study Group. ‘Long-COVID’: A cross-sectional study of persisting symptoms, biomarker and imaging abnormalities following hospitalisation for COVID-19. Thorax.

[8] Coronavirus Disease 2019 (COVID-19): Post COVID-19 Condition. https://www.who.int/news-room/questions-and-answers/item/coronavirus-disease-(covid-19)-post-covid-19-condition.

[9] Cabrera Martimbianco A.L., Pacheco R.L., Bagattini Â.M., Riera R. (2021). Frequency, signs and symptoms, and criteria adopted for long COVID-19: A systematic review. Int. J. Clin. Pract..

[10] Crook H., Raza S., Nowell J., Young M., Edison P. (2021). Long covid-mechanisms, risk factors, and management. BMJ.

[11] Yong S.J. (2021). Long COVID or post-COVID-19 syndrome: Putative pathophysiology, risk factors, and treatments. Infect. Dis..

[12] Ceban F., Ling S., Lui L.M.W., Lee Y., Gill H., Teopiz K.M., Rodrigues N.B., Subramaniapillai M., Di Vincenzo J.D., Cao B. (2022). Fatigue and cognitive impairment in Post-COVID-19 Syndrome: A systematic review and meta-analysis. Brain. Behav. Immun..

[13] Kujawski S., Zalewski P., Newton J.L. (2021). Do some long COVID patients suffer from ME/CFS?. Med. Res. J..

[14] Renaud-Charest O., Lui L.M.W., Eskander S., Ceban F., Ho R., Di Vincenzo J.D., Rosenblat J.D., Lee Y., Subramaniapillai M., McIntyre R.S. (2021). Onset and frequency of depression in post-COVID-19 syndrome: A systematic review. J. Psychiatr. Res..

[15] Vindegaard N., Benros M.E. (2020). COVID-19 pandemic and mental health consequences: Systematic review of the current evidence. Brain. Behav. Immun..

[16] Kubica A., Michalski P., Kasprzak M., Podhajski P., Pietrzykowski Ł., Rzepka-Cholasińska A., Fabiszak T., Kryś J. (2021). Functioning of patients with post-COVID syndrome—preliminary data. Med. Res. J..

[17] Winzer E.B., Woitek F., Linke A. (2018). Physical Activity in the Prevention and Treatment of Coronary Artery Disease. J. Am. Heart. Assoc..

[18] Soderlund P.D. (2018). Effectiveness of motivational interviewing for improving physical activity self-management for adults with type 2 diabetes: A review. Chronic Illn..

[19] Navidad L., Padial-Ruz R., González M.C. (2021). Nutrition, Physical Activity, and New Technology Programs on Obesity Prevention in Primary Education: A Systematic Review. Int. J. Environ. Res. Public Health.

[20] Michalski P., Kosobucka A., Pietrzykowski Ł., Kasprzak M., Buszko K., Obońska K., Fabiszak T., Kubica A. (2018). Effectiveness of therapeutic education in patients with myocardial infarction. Med. Res. J..

[21] Michalski P., Kasprzak M., Siedlaczek M., Kubica A. (2020). The impact of knowledge and effectiveness of educational intervention on readiness for hospital discharge and adherence to therapeutic recommendations in patients with acute coronary syndrome. Med. Res. J..

[22] Peçanha T., Goessler K.F., Roschel H., Gualano B. (2020). Social isolation during the COVID-19 pandemic can increase physical inactivity and the global burden of cardiovascular disease. Am. J. Physiol. Heart. Circ. Physiol..

[23] Rinaldo R.F., Mondoni M., Parazzini E.M., Pitari F., Brambilla E., Luraschi S., Balbi M., Sferrazza Papa G.F., Sotgiu G., Guazzi M. (2021). Deconditioning as main mechanism of impaired exercise response in COVID-19 survivors. Eur. Respir. J..

[24] Kubica A., Michalski P., Pietrzykowski Ł., Rzepka-Cholasińska A., Kosobucka-Ozdoba A., Jasiewicz M., Laskowska E., Kryś J., Ostrowska M. (2022). Post-COVID-19 rehabilitation (PCR-SIRIO 8) study. A rationale and protocol of the study. Med. Res. J..

[25] Kuodi P., Gorelik Y., Zayyad H., Wertheim O., Wiegler K.B., Jabal K.A., Dror A.A., Nazzal S., Glikan D., Edelstein M. (2022). Association between vaccination status and reported incidence of post-acute COVID-19 symptoms in Israel: A cross-sectional study 2020-21, Israel. NPJ Vaccines.

[26] Antonelli M., Penfold R.S., Merino J., Sudre C.H., Molteni E., Berry S., Canas L.S., Graham M.S., Klaser K., Modat M. (2022). Risk factors and disease profile of post-vaccination SARS-CoV-2 infection in UK users of the COVID Symptom Study app: A prospective, community-based, nested, case-control study. Lancet Infect. Dis..

[27] (2021). COVID-19 Rapid Guideline: Managing the Long-Term Effects of COVID-19.

[28] Barker-Davies R.M., O’Sullivan O., Senaratne K.P.P., Baker P., Cranley M., Dharm-Datta S., Ellis H., Goodall D., Gough M., Lewis S. (2020). The Stanford Hall consensus statement for post-COVID-19 rehabilitation. Br. J. Sports. Med..

[29] Liu K., Zhang W., Yang Y., Zhang J., Li Y., Chen Y. (2020). Respiratory rehabilitation in elderly patients with COVID-19: A randomized controlled study. Complement. Ther. Clin. Pract..

[30] Hermann M., Pekacka-Egli A.M., Witassek F., Baumgaertner R., Schoendorf S., Spielmanns M. (2020). Feasibility and Efficacy of Cardiopulmonary Rehabilitation After COVID-19. Am. J. Phys. Med. Rehabil..

[31] Takekawa T., Kashiwabara K., Yamada N., Watanabe S., Hama M., Hashimoto G., Abo M., Shinfuku K. (2022). Rehabilitation therapy for a severe case of coronavirus disease 2019: A case report. J. Med. Case Rep..

[32] Udina C., Ars J., Morandi A., Vilaró J., Cáceres C., Inzitari M. (2021). Rehabilitation in adult post-COVID-19 patients in post-acute care with Therapeutic Exercise. J. Frailty Aging.

[33] Barbara C., Clavario P., De Marzo V., Lotti R., Guglielmi G., Porcile A., Russo C., Griffo R., Mäkikallio T., Hautala A.J. (2022). Effects of exercise rehabilitation in patients with long coronavirus disease 2019. Eur. J. Prev. Cardiol..

[34] Besnier F., Bérubé B., Malo J., Gagnon C., Grégoire C.A., Juneau M., Simard F., L’Allier P., Nigam A., Iglésies-Grau J. (2022). Cardiopulmonary Rehabilitation in Long-COVID-19 Patients with Persistent Breathlessness and Fatigue: The COVID-Rehab Study. Int. J. Environ. Res. Public Health.

[35] Morrow A., Gray S.R., Bayes H.K., Sykes R., McGarry E., Anderson D., Boiskin D., Burke C., Cleland J.G.F., Goodyear C. (2022). Prevention and early treatment of the long-term physical effects of COVID-19 in adults: Design of a randomised controlled trial of resistance exercise-CISCO-21. Trials.

[36] Flannery T., Brady-Sawant H., Tarrant R., Davison J., Shardha J., Halpin S., Sivan M., Ross D. (2022). A Mixed-Methods Evaluation of a Virtual Rehabilitation Program for Self-Management in Post-COVID-19 Syndrome (Long COVID). Int. J. Environ. Res. Public Health.

